# DNA methylation and gene expression patterns in breast cancer progression from *in situ* carcinoma to invasive carcinoma

**DOI:** 10.1186/1756-8935-6-S1-P18

**Published:** 2013-03-18

**Authors:** Thomas Fleischer, Hege Edvardsen, Jovana Jovanovic, Nizar Touleimat, Anne-Lise Børresen-Dale, Jörg Tost, Vessela N Kristensen

**Affiliations:** 1 Department of Genetics, Institute for Cancer Research, Oslo University Hospital Radiumhospitalet, Oslo, Norway; 2 The K.G. Jebsen Center for Breast Cancer Research, Institute for Clinical Medicine, Faculty of Medicine, University of Oslo, Norway; 3 Institute for Clinical Epidemiology and Molecular Biology (EpiGen), Faculty of Medicine, University of Oslo, Norway; 4 Laboratory for Epigenetics, Centre National de Génotypage, CEA - Institut de Génomigue, 91000 Evry France

## Background

Breast cancer is a disease caused by uncontrolled cell division of epithelial cells in the ducts or the lobules of the breast. The ducts and lobules are enclosed by a basement membrane, and during progression of the disease the invading cells will breach the membrane and invade adjacent tissue. A tumor that is still enclosed in the basement membrane is called a carcinoma *in situ*, while a tumor that has breached the basement membrane is called an invasive carcinoma. DNA methylation is a DNA modification where methyl groups are added to CpG dinucleotides and thought to regulate gene expression through blocking of transcription factor binding or through chromatin remodeling. The aim of the study was to determine what genes get differentially methylated when the cancer progresses from a less to a more aggressive carcinoma. In addition, by applying integrated analysis of other molecular data such as gene expression and copy number, we could investigate how more elaborate biological processes change during progression. Being able to determine the processes that take place during progression of breast cancer may give valuable insight into cancer biology, as well as identification of early markers of disease.

## Materials and methods

Methylation status of 239 tumors and 46 healthy tissue controls were determined by lllumina Infinium 450K methylation array, interrogating about 480 000 CpGs distributed in promoters, gene bodies, 3’UTRs and intergenic regions. Gene expression, miRNA expression, array CGH and clinical parameters are available for a subset of samples. The samples were collected at hospitals in Oslo/Akershus, Norway, and all patients have given informed consent and the projects are approved by the local ethical committee.

## Results

Results that will be presented include differentially methylated genes between healthy breast tissue with varying mammographic density, differentially methylated genes between healthy tissue and carcinoma *in situ* and differentially methylated genes between carcinoma *in situ* and early stage invasive carcinoma. Methylation level of CpGs in many genes showed differential methylation when comparing *in situ* carcinoma and invasive carcinoma. The top hits include *CTNNA1*, *FAIM2*, *MCAM*, *PDZK1*, *PLAT*, *PPP2R1B* and *PSG3 -* genes that are involved in cell differentiation, growth and adhesion. Also investigated was the association between DNA methylation and gene expression, with focus on CpG position relative to transcription start site. Preliminary results indicate that almost a third of significant hits show a positive correlation between methylation and expression. CpGs with positive correlation to gene expression were most often found in gene bodies and 3’UTRs.These findings challenge the traditional view that DNA methylation can only inhibit gene expression.

## Conclusions

We observe changes in DNA methylation patterns between tissues with varying stage of progression in breast cancer and we report both positive and negative correlation between DNA methylation and gene expression.

